# Editorial: Surgical oncology in the elderly: the state of the art and future challenges, volume II

**DOI:** 10.3389/fonc.2025.1544123

**Published:** 2025-02-07

**Authors:** Lucia Moletta, Felix Berlth, Evangelos Tagkalos, Cosimo Sperti, Giovanni Capovilla

**Affiliations:** ^1^ Department of Surgery, Oncology and Gastroenterology, 1st Surgical Clinic, University of Padua, Padova, Italy; ^2^ Klinik für Allgemein-, Viszeral- und Transplantationschirurgie, Universitätsklinikum Tübingen, Eberhard Karls Universität, Tübingen, Germany; ^3^ Department of Visceral Surgery and Transplantation, Mainz University, Johannes Gutenberg Universitat Mainz, Mainz, Germany; ^4^ Department of Surgery, Oncology and Gastroenterology, Hepato-Bilio-Pancreatic (HPB) Unit, University of Padua, Padova, Italy

**Keywords:** surgical oncology, elderly, esophageal cancer, gastric cancer, pancreatic cancer, colorectal cancer

Surgical oncology in older adults involves distinct challenges and factors, primarily due to the intricate interactions between age-related physiological changes, existing comorbidities, and modified cancer biology (1). Elderly patients typically have diminished physiological reserves, which can affect their capacity to endure major surgeries and recover effectively afterward. Furthermore, age-related reductions in organ function, such as decreased renal or hepatic clearance, may influence the metabolism of anesthetics and medications (2). The presence of comorbidities, including cardiovascular disease, diabetes, and frailty, adds complexity to surgical decision-making. Despite these obstacles, surgery continues to be a key component of cancer treatment for elderly patients, provided that proper risk assessment and personalized treatment plans are in place. Considering the unique characteristics of the elderly and the growing number of older cancer patients due to population aging, it is crucial to identify appropriate perioperative pathways tailored specifically to these patients. As a matter of fact, research indicates that, with careful patient selection and a multidisciplinary approach, elderly patients can achieve outcomes similar to younger individuals, especially when the tumors are localized and amenable to surgery (3). Advances in minimally invasive techniques, enhanced recovery protocols, and better perioperative care are helping to improve surgical outcomes and quality of life for elderly cancer patients (4, 5). Similarly, early identification of frailty enables tailored perioperative management, such as optimization of nutritional status, physical conditioning, and managing comorbidities, which can improve surgical outcomes and enhance postoperative recovery. Frailty (defined as a state of decreased physiological reserve) is a critical factor in the evaluation of elderly patients undergoing oncologic surgery, as it is strongly associated with poor surgical outcomes, including increased complications, longer hospital stays, and higher mortality rates (6, 7). Frailty assessment can guide decision-making in terms of treatment strategies, offering a more personalized approach to care in elderly cancer patients. Similarly, analysis of quality-of-life (QoL) metrics and functional outcomes in elderly patients undergoing major surgery is crucial for understanding the broader implications of surgical interventions, particularly in oncological settings. Elderly patients may face a range of challenges beyond survival, such as maintaining independence, coping with physical and cognitive changes, and managing comorbidities. In this context, QoL and functional outcomes serve as essential indicators of a patient’s overall well-being and recovery.

This Research Topic have focused on the surgical management of solid malignancies in elderly patients, addressing the preoperative work-up, the role of prognostic factors and indicators of frailty and the impact of age on short and long-term surgical outcomes.

The manuscript by Friziero et al. reports a retrospective case series of octogenarian patients undergoing emergency operations for oncological acute illnesses. The Authors analyzed the postoperative outcomes of 102 patients who were divided into two groups: the octogenarian group (n = 42; patients aged ≥ 80) and the younger patients (n = 60; patients aged < 80). Thirty-day mortality and 90-day mortality was similar between the two groups. Similarly, postoperative complications and length of hospital stay were not significantly different in the two groups. These manuscript highlights that age alone does not represent an independent risk factor for poor outcomes in surgical oncological emergencies. Therefore, octogenarians should not be excluded *a priori* from surgical options in cases of oncological emergencies, provided they have acceptable clinical conditions.

Hu et al. report a rare case of gastric paraganglioma (PGL) in an elderly patient. Gastric PGLs are extremely uncommon, with a five-year survival rate of less than 5%, and there is limited literature on this clinical entity. The Authors describe the case of a 72-year-old man complaining persistent left upper abdominal pain and anemia. Endoscopic and radiological exams revealed a tumor in the gastric antrum, with pathology suggesting adenocarcinoma. The patient was treated with neoadjuvant chemotherapy, showing a partial response, and then underwent radical gastrectomy with concurrent D2 lymph node dissection. Notably, the patient’s blood pressure fluctuated during surgery, reaching as high as 240 mmHg. Histological examination confirmed the diagnosis of gastric paraganglioma, with one regional lymph node metastasis. This case report underlines how rare histologies can be found in elderly patients, and that major oncologic surgery can be proposed, provided an adequate preoperative functional study of the patient is conducted.

Zhang et al. analyzed the differences in the efficacy and safety of radical cystectomy with ileal conduit across age subgroups to assess the impact of age on the procedure. Patients were divided into two groups: elderly (≥70 years at diagnosis) and non-elderly (<70 years). For external validation, data from 416 patients in the SEER database were examined, with 172 categorized as non-elderly and 244 as elderly. The study found that elderly patients were more likely to require ICU transfer postoperatively but had a lower incidence of stoma inflammation, with a similar efficacy of radical cystectomy with ileal conduit when compared to non-elderly patients. This study emphasizes once again that, although there are surgical and perioperative risks in elderly patients, old age should not be used as an absolute exclusion criterion for major oncologic surgery.

Huang et al. investigated the impact of lymph node involvement on overall survival in elderly patients with non-metastatic gallbladder adenocarcinoma. They analyzed data from 1,654 patients, of which 706 had lymph node involvement and 948 did not. The study identified several risk factors for prognosis, including age, tumor size, lymph node involvement, gender, and surgical treatment. A nomogram was developed to assess the prognostic impact at 1, 3, and 5 years. Overall survival (OS) was significantly worse for patients undergoing surgery with nodal involvement (hazard ratio [HR], 2.238; P < 0.01). Additionally, after adjustment, the presence of more than two metastatic lymph nodes was associated with a further decrease in OS (HR, 3.305; P < 0.01). The authors concluded that lymph node involvement is linked to poorer survival in elderly patients. A threshold of more than two metastatic lymph nodes appears to have significant prognostic value, suggesting the need for closer monitoring of elderly patients with gallbladder cancer who have multiple lymph node involvements, and highlighting the importance of a multidisciplinary, tailored approach for this particular subset of patients.

Based on the studies reviewed, several actionable recommendations for clinical practice and future research can be proposed:

1. Early Identification of Frailty: Implementing routine frailty assessments in elderly cancer patients is essential for guiding perioperative management. Frailty markers such as physical performance, nutrition, and comorbidities should be considered when determining surgical eligibility and postoperative care plans.
2. Personalized Treatment Strategies: The studies suggest that with careful patient selection, major cancer surgeries can be safe for elderly patients. However, treatment plans should be individualized, considering both cancer biology and the patient’s overall health, including frailty and comorbid conditions.
3. Enhancing Quality-of-Life Assessments: Future studies should incorporate QoL and functional outcomes to provide a more comprehensive evaluation of surgical interventions in elderly cancer patients. This will allow clinicians to better understand the long-term effects of surgery on the patient’s well-being and independence.
4. Multidisciplinary Approach: A multidisciplinary approach involving geriatricians, oncologists, surgeons, and rehabilitation specialists should be standard practice in managing elderly cancer patients, ensuring that all aspects of the patient’s health are considered in decision-making.

This Research Topic underscores the potential for successful cancer surgery in older adults, provided that individualized, evidence-based strategies are employed. Although age alone is not a contraindication for surgery, frailty and comorbidities play a pivotal role in determining surgical outcomes. By incorporating frailty assessments, personalized treatment plans, and a focus on postoperative functional recovery and QoL, the surgical management of elderly cancer patients can be optimized, improving both survival and quality of life.
